# Supplementary material for: Effects of prophylactic nebulized antibiotics on the prevention of ICU-acquired pneumonia: a systematic review and meta-analysis
Source: PeerJ. 2024 Dec 13;12:e18686. doi: 10.7717/peerj.18686 (PMC11648687; doi:10.7717/peerj.18686)

Effect of prophylactic nebulized antibiotics in the prevention of ICU-acquired pneumonia: A systematic review and meta-analysis

**Appendix files**

Appendix 1 Search Strategy…………………………………………………………………………………………………..……………………..2

Appendix 2 Fig S1 Risk of bias ………………………………………………………………………………………………………..…………..7

Appendix 3 Tests for publication bias for RR of ICU-acquired pneumonia ….…………………………………………..……8

Appendix 4 Percentages of items of included articles that produced risks of bias …………………………………..……9

Appendix 5 GRADE Assessment of meta-analytic results ………………………………………………………………………..…10

Appendix 6 Sensitivity analysis of different endpoints …………………………………………………………..……….…...11

Appendix 7 Forest plots of the effects of prophylactic nebulized antibiotics on the secondary outcomes……………………………………………………………………………………………………………………………………..……….…...13

**Appendix 1 Search Strategy** (Limited to RCT）

Database: PubMed，Embase，Cochrane library

----------------------------------------------------------------------------------------------------------------------

**PubMed**

#1 "Aerosols"[Mesh]

#2 "Administration, Inhalation"[Mesh]

#3 "Nebulizers and Vaporizers"[Mesh]

#4 nasal sprays[Title/Abstract]

#5 oral sprays[Title/Abstract]

#6 Drug Administration, Respiratory[Title/Abstract]

#7 Administration, Respiratory Drug[Title/Abstract]

#8 Respiratory Drug Administration[Title/Abstract]

#9 Inhalation Drug Administration[Title/Abstract]

#10 Drug Administration, Inhalation[Title/Abstract]

#11 Administration, Inhalation Drug[Title/Abstract]

#12 Inhalation Administration[Title/Abstract]

#13 Drug Aerosol Therapy[Title/Abstract]

#14 Aerosol Therapy, Drug[Title/Abstract]

#15 Therapy, Drug Aerosol[Title/Abstract]

#16 Drug Therapy, Aerosol[Title/Abstract]

#17 Aerosol Drug Therapy[Title/Abstract]

#18 Therapy, Aerosol Drug[Title/Abstract]

#19 Inhalation of Drugs[Title/Abstract]

#20 Inhalation Device[Title/Abstract]

#21 Devices, Inhalation[Title/Abstract]

#22 Device, Inhalation[Title/Abstract]

#23 Inhalation Devices[Title/Abstract]

#24 Inhalation Device[Title/Abstract]

#25 Atomizer[Title/Abstract]

#26 Atomizers[Title/Abstract]

#27 Nebulizer[Title/Abstract]

#28 Nebulizers[Title/Abstract]

#29 Inhalator[Title/Abstract]

#30 Inhalators[Title/Abstract]

#31 Inhaler[Title/Abstract]

#32 Inhalers[Title/Abstract]

#33 Vaporizer[Title/Abstract]

#34 Vaporizers[Title/Abstract]

#35 "Healthcare-Associated Pneumonia"[Mesh]

#36 "Pneumonia, Ventilator-Associated"[Mesh]

#37 HAP[Title/Abstract]

#38 VAP[Title/Abstract]

#39 ventilator-associated pneumonia[Title/Abstract]

#40 ICU-acquired pneumonia[Title/Abstract]

#41 Healthcare Associated Pneumonia[Title/Abstract]

#42 Healthcare-Associated Pneumonias[Title/Abstract]

#43 Pneumonia, Healthcare-Associated[Title/Abstract]

#44 Nosocomial Pneumonia[Title/Abstract

#45 Nosocomial Pneumonias[Title/Abstract

#46 Pneumonia, Nosocomial[Title/Abstract]

#47 Hospital Acquired Pneumonia[Title/Abstract]

#48 Hospital Acquired Pneumonias[Title/Abstract

#49 Pneumonia, Hospital Acquired[Title/Abstract]

#52 randomized controlled trial[Publication Type]

#53 controlled clinical trial[Publication Type]

#54 randomized[Title/Abstract]

#55 placebo[Title/Abstract]

#56 randomly[Title/Abstract]

#57 groups[Title/Abstract]

#58 trial[Publication Type]

（#1 OR #2 OR #3 OR #4 OR #5 OR #6 OR #7 OR #8 OR #9 OR #10 OR #11 OR #12 OR #13 OR #14 OR #15 OR #16 OR #17 OR #18 OR #19 OR #20 OR #21 OR #22 OR #23 OR #24 OR #25 OR #26 OR #27 OR #28 OR #29 OR #30 OR #31 OR #32 OR #33 OR #34 ）AND（#35 OR #36 OR #37 OR #38 OR #39 OR #40 OR #41 OR #42 OR #43 OR #44 OR #45 OR #46 OR #47 OR #48 OR #49 ）AND (#52 OR #53 OR #54 OR #55 OR #65 OR #7 OR #58 )

----------------------------------------------------------------------------------------------------------------------

**Embase**

#16 "healthcare associated pneumonias':ab,ti

#15 ventilator-associated pneumonias":ab,ti

#14 'icu-acquired pneumonias':ab,ti

#13 "hospital acquired pneumonias':ab,ti

#12 "hospital acquired pneumonia':ab,ti

#11 'nosocomial pneumonias':ab,ti

#10 'nosocomial pneumonia':ab,ti

#9 "healthcare-associated pneumonia':ab,ti

#8 "healthcare-associated pneumonias':ab,ti

#7 "healthcare associated pneumonia':ab,ti

#6 "icu-acquired pneumonia':ab,ti

#5 'ventilator-associated pneumonia':ab,ti

#4 vap':ab.ti

#3 'hap':ab.ti

#2 'ventilator associated pneumonia'/exp

#1 'health care associated pneumonia'/exp

#17 #1 OR#2 OR#3 OR#4 OR#5 OR#6 OR#7 OR#8 OR#9 OR#10 OR#11 OR#12 OR#13 OR#14 OR#15 OR#16

#42 'vaporizer*":ab,ti

#41 'inhaler*":ab,ti

#40 'inhalator*":ab,ti

#39 'nebulizer*":ab,ti

#38 'atomizer**:ab,ti

#37 "inhalation device*":ab,ti

#36 'inhalation of drugs':ab,ti

#35 'therapy aerosol drug':ab,ti

#34 'aerosol drug therapy':ab,ti

#33 'drug therapy aerosol':ab,ti

#32 'therapy drug aerosol':ab,ti

#31 'aerosol therapy drug':ab,ti

#30 'drug aerosol therapy':ab,ti

#29 'inhalation administration':ab,ti

#28 'administration inhalation drug':ab,ti

#27 'drug administration inhalation':ab,ti

#26 "inhalation drug administration':ab,ti

#25 'respiratory drug administration':ab,ti

#24 'administration respiratory drug':ab,ti

#23 'drug administration respiratory':ab,ti

#22 'oral sprays':ab,ti

#21'nasal sprays':ab,ti

#20 'nebulizer'/exp

#19 'inhalational drug administration'/exp

#18 'aerosol'/exp

#43 #18 OR#19 OR#20 OR#21 OR#22 OR#23 OR#24 OR#25 OR#26 OR#27 OR#28 OR#29 OR#30 OR#31 OR#32 OR#33 OR #34 OR#35 OR#36 OR#37 OR#38 OR #39 OR#40 OR#41 OR#42

#47 'clinical trial/exp

#46 'placebo'/exp

#45 'controlled clinical trial'/exp

#44 'randomized controlled trial'/exp

#48 #44 OR#45 OR#46 OR#47

#49 #17 AND#43 AND #48

----------------------------------------------------------------------------------------------------------------------

**Cochrane library**

#1 MeSH descriptor: [Aerosols] explode all trees

#2 MeSH descriptor: [Inhalation] explode all trees

#3 MeSH descriptor: [Nebulizers and Vaporizers] explode all trees

#4 (nebul*):ti,ab,kw (Word variations have been searched)

#5 (aerosol*):ti,ab,kw (Word variations have been searched)

#6 (vaporiz*):ti,ab,kw (Word variations have been searched)

#7 (inhal*):ti,ab,kw (Word variations have been searched)

#8 (pulmonary delivery*):ti,ab,kw (Word variations have been searched)

#9 (atomiz*):ti,ab,kw (Word variations have been searched)

#10 #1 or #2 or #3 or #4 or #5 or #6 or #7 or #8 or #9

#11 MeSH descriptor: [Healthcare-Associated Pneumonia] explode all trees

#12 MeSH descriptor: [Pneumonia, Ventilator-Associated] explode all trees

#13 (HAP):ti,ab,kw (Word variations have been searched)

#14 (VAP):ti,ab,kw (Word variations have been searched)

#15 (ventilator-associated pneumonia):ti,ab,kw (Word variations have been searched)

#16 (ICU-acquired pneumonia):ti,ab,kw (Word variations have been searched)

#17 (Healthcare Associated Pneumonia*):ti,ab,kw (Word variations have been searched)

#18 (Healthcare-Associated Pneumonia*):ti,ab,kw (Word variations have been searched)

#19 (Pneumonia, Healthcare-Associated):ti,ab,kw (Word variations have been searched)

#20 (nosocomial pneumonia*):ti,ab,kw (Word variations have been searched)

#21 (Pneumonia, Nosocomial):ti,ab,kw (Word variations have been searched)

#22 (Hospital Acquired Pneumonia*):ti,ab,kw (Word variations have been searched)

#23 (Pneumonia, Hospital Acquired):ti,ab,kw (Word variations have been searched)

#24 (viral respiratory infection*):ti,ab,kw (Word variations have been searched)

#25 (lung infection*):ti,ab,kw (Word variations have been searched)

#26 (ventilator associated pneumonia*):ti,ab,kw (Word variations have been searched)

#27 #11 or #12 or #13 or #14 or #15 or #16 or #17 or #18 or #19 or #20 or #21 or #22 or #23 or #24 or #25 or #26

#28 #10 and #27

----------------------------------------------------------------------------------------------------------------------

**Studies needed for full-reviewed but not included in the current meta-analysis (n=12 trials)**

[1] Khubutiya M, Shabanov AK, Chernen'Kaya TV, et al. Early administration of inhaled tobramycin in patients with severe multiple trauma. Pulmonologiya, 2015, 25(2): 211‐216.

[2] Kuzovlev A, Shabanov A, Chernenkaya T, et al. Early preventive administration of inhaled tobramycin in severe polytrauma. Critical care (London, England), 2015, 19: S42.

[3] Palmer LB, Smaldone GC. Reduction of bacterial resistance with inhaled antibiotics in the intensive care unit. American journal of respiratory and critical care medicine, 2014, 189(10): 1225‐1233.

[4] Klick JM, du Moulin GC, Hedley-Whyte J, et al. Prevention of gram-negative bacillary pneumonia using polymyxin aerosol as prophylaxis. II. Effect on the incidence of pneumonia in seriously ill patients. J Clin Invest, 1975, 55(3): 514-519.

[5] Hullard-Pulstinger A, Holler E, Hahn J, et al. Prophylactic application of nebulized liposomal amphotericin B in hematologic patients with neutropenia. Onkologie, 2011, 34(5): 254-258.

[6] Golden JA, Katz MH, Chernoff DN, et al. A randomized comparison of once-monthly or twice-monthly high-dose aerosolized pentamidine prophylaxis. Chest, 1993, 104(3): 743-750.

[7] Reichenspurner H, Gamberg P, Nitschke M, et al. Significant reduction in the number of fungal infections after lung-, heart-lung, and heart transplantation using aerosolized amphotericin B prophylaxis. Transplant Proc, 1997, 29(1-2): 627-628.

[8] Tavernier E, Barbier F, Meziani F, et al. Inhaled amikacin versus placebo to prevent ventilator-associated pneumonia: the AMIKINHAL double-blind multicentre randomised controlled trial protocol. BMJ Open, 2021, 11(9): e048591.

[9] Drew RH, Dodds Ashley E, Benjamin DK, Jr., et al. Comparative safety of amphotericin B lipid complex and amphotericin B deoxycholate as aerosolized antifungal prophylaxis in lung-transplant recipients. Transplantation, 2004, 77(2): 232-237.

[10] van der Hoeven SM, Binnekade JM, de Borgie CA, et al. Preventive nebulization of mucolytic agents and bronchodilating drugs in invasively ventilated intensive care unit patients (NEBULAE): study protocol for a randomized controlled trial. Trials, 2015, 16: 389.

[11] Falagas ME, Siempos, II, Bliziotis IA, et al. Administration of antibiotics via the respiratory tract for the prevention of ICU-acquired pneumonia: a meta-analysis of comparative trials. Crit Care, 2006, 10(4): R123.

[12] Póvoa FCC, Cardinal-Fernandez P, Maia IS, et al. Effect of antibiotics administered via the respiratory tract in the prevention of ventilator-associated pneumonia: A systematic review and meta-analysis. Journal of Critical Care, 2018, 43: 240-245.

**Appendix 2**

Fig. S1 Risk of bias summary


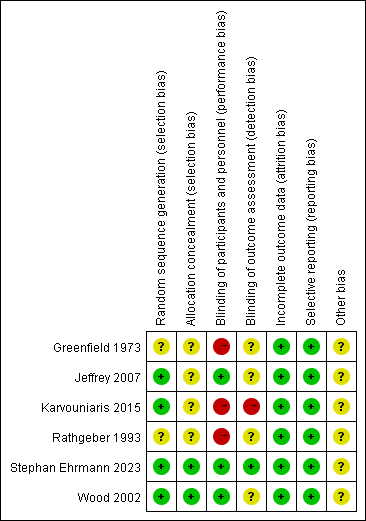


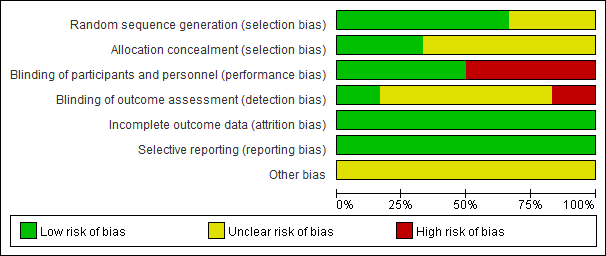


**Appendix 3**

Fig.S2a Tests for publication bias for RR of ICU-acquired pneumonia.


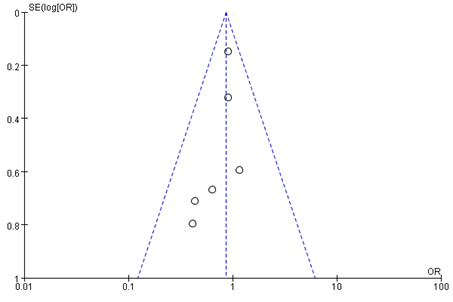


Fig. S2b Tests for publication bias for RR of ICU-acquired pneumonia by egger's test (P=0.172)


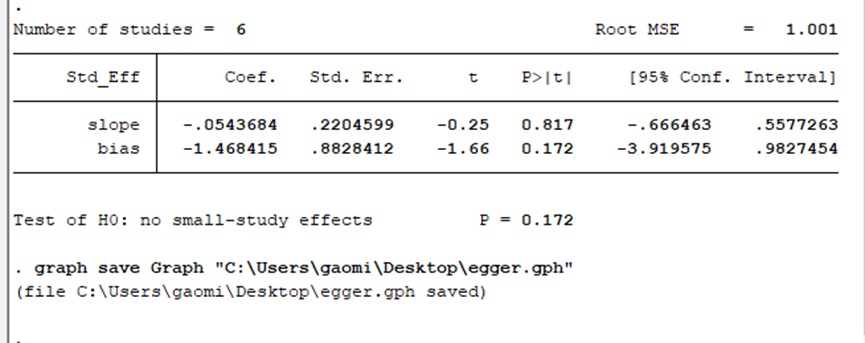


ICU: intensive care unit;

**Appendix 4 Percentages of items of included articles that produced risks of bias**

Fig.S3 Risk of bias assessment


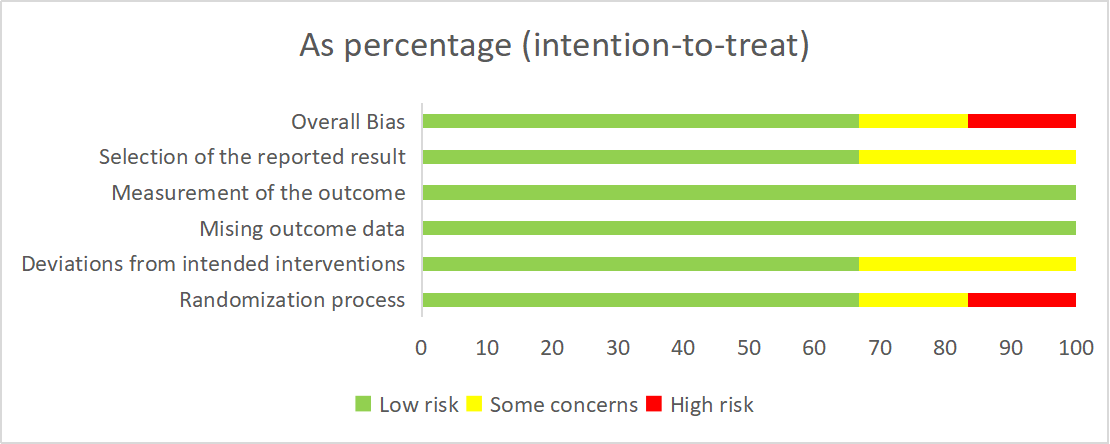


**Appendix 5** GRADE Assessment of meta-analytic results

Fig.S4 GRADE Assessment of meta-analytic results


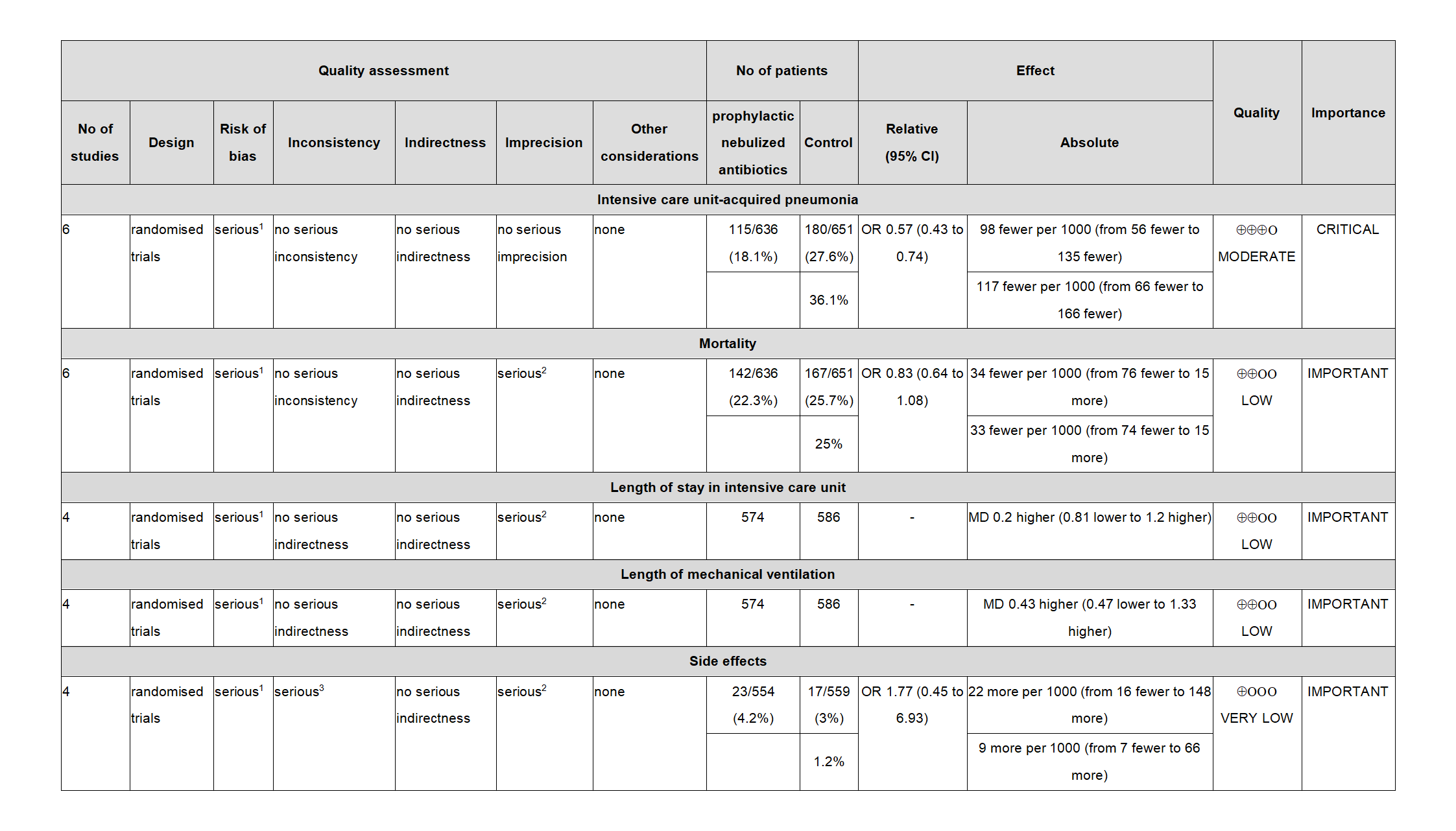


^1^ bigger bias in terms of randomization, allocation concealment, and blinding methods
^2^  Passing through equivalent lines

^3^  I^2^ 50-75

**Appendix 6**  Sensitivity analysis of different endpoints

Fig.S5a Sensitivity analysis of intensive care unit-acquired pneumonia.


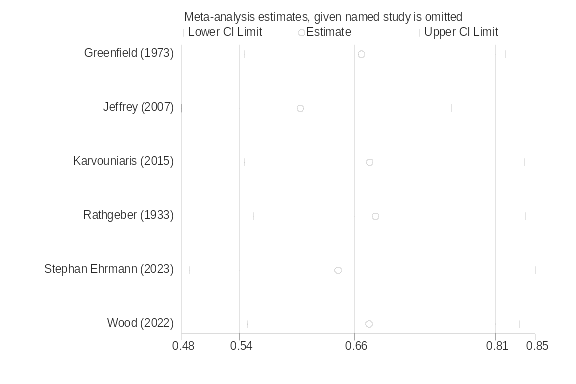


Fig.S5b Sensitivity analysis of the length of stay in intensive care unit.

Fig.S5c Sensitivity analysis of duration of mechanical ventilation.

Fig.S5d Sensitivity analysis of mortality.

Fig.S5e Sensitivity analysis of the side effects of prophylactic nebulized antibiotics.

**Appendix 7 Forest plots of the effects of prophylactic nebulized antibiotics on the secondary outcomes**

Fig.S6 Forest plots of the effects of prophylactic nebulized antibiotics on the length of stay in intensive care unit.


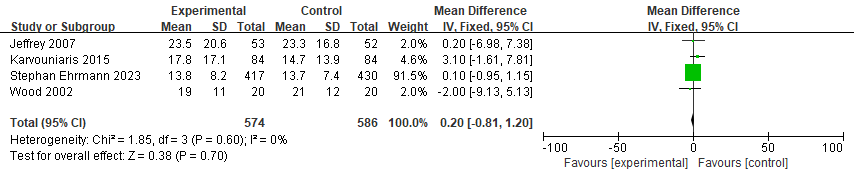


Fig.S7 Forest plots of the effects of prophylactic nebulized antibiotics on duration of mechanical ventilation.


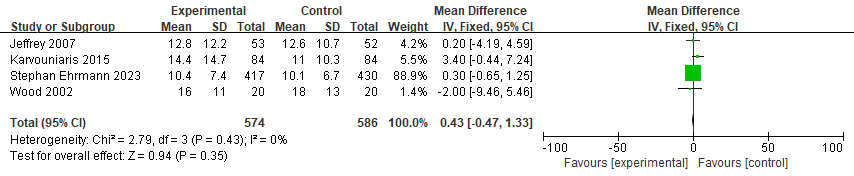


Fig.S8 Forest plots of the side effects of prophylactic nebulized antibiotics.


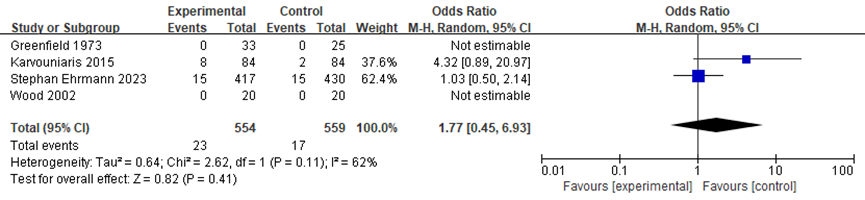

Supplement: Supplemental Information 1 [file peerj-12-18686-s001.doc]
